# Ultrasonographic fetal sex determination in large domestic animals: a comparative, mechanistic, and field-oriented synthesis

**DOI:** 10.3389/fvets.2026.1867201

**Published:** 2026-06-17

**Authors:** Ahmed Ali, Derar R. Derar, Yousef M. Alharbi

**Affiliations:** 1 Department of Clinical Sciences, College of Veterinary Medicine, Qassim University, Buraydah, Saudi Arabia; 2 Department of Medical Biosciences, College of Veterinary Medicine, Qassim University, Buraydah, Saudi Arabia

**Keywords:** comparative analysis, diagnostic accuracy, fetal sex determination, genital tubercle, large domestic animals, livestock reproduction, reproductive management, ultrasonography

## Abstract

**Background:**

Ultrasonographic fetal sex determination is commonly used in livestock production to aid genetic selection, reproductive planning, and economic optimization. Despite consistently high diagnostic accuracies under controlled conditions, its use in field settings is variable and frequently unpredictable. This disparity is due to the combined effects of embryological development, species-specific anatomy, fetal positioning, operator expertise, and technical limitations.

**Objective:**

This review aims to provide a comparative and integrative synthesis of ultrasonographic fetal sex determination across major domestic species, bridging the gap between experimental performance and field applicability.

**Methods:**

A comprehensive analysis was conducted, combining published literature with extensive field experience with cattle, camels, horses, and buffaloes. The review is organized around three key dimensions: (i) biological timing of sexual differentiation, (ii) diagnostic visibility of ultrasonographic landmarks, and (iii) practical feasibility in field conditions.

**Results:**

Across species, the genital tubercle is the primary diagnostic landmark during early gestation; however, its visibility and diagnostic reliability vary depending on the species' developmental dynamics and imaging accessibility. One important finding is the distinction between diagnostic accuracy and diagnostic feasibility, with the latter emerging as the primary limitation in field conditions. According to comparative analysis, cattle provide the most consistent environment for early diagnosis, horses provide the broadest diagnostic window, camels present a narrow but precise window, and buffaloes are relatively under-characterized.

**Conclusion:**

Ultrasonographic fetal sex determination is a highly accurate but context-dependent tool, with success determined more by feasibility than diagnostic capability. The integration of emerging technologies, such as Doppler ultrasonography, three-dimensional imaging, and artificial intelligence, has the potential to improve consistency and reduce operator dependency. This review creates a unified framework that connects embryology, imaging, and field application, offering practical advice and defining priorities for future research in livestock reproduction.

## Graphical Abstract

**Figure:**
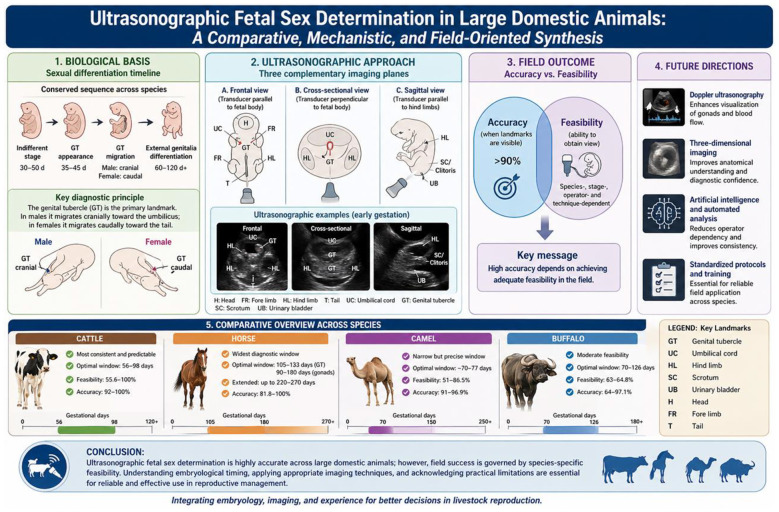
Graphical abstract summarizing the biological basis, ultrasonographic approaches, comparative species performance, and future directions of fetal sex determination in large domestic animals. The schematic integrates embryological development, diagnostic imaging planes, and field applicability, emphasizing the relationship between accuracy and feasibility across species.

## 1 Introduction

Prenatal fetal sex determination in large domestic animals has progressed from a research technique to a useful tool with significant economic and managerial implications (1–4). Early detection of fetal sex enables producers to align breeding strategies with market demand, optimize replacement programs, and boost herd productivity (2, 5, 6). Ultrasonography has become the preferred method for veterinary practice due to its non-invasive nature, real-time imaging capability, and widespread availability (4, 5, 7–12).

Despite these advantages, there is a persistent disparity between the high diagnostic accuracies reported in experimental studies and the variable success rates observed in field settings. This inconsistency is most noticeable when comparing different species, gestational stages, and operator skill levels (13, 14). While most studies show accuracies greater than 90% within optimal windows, practitioners frequently face limitations due to fetal position, anatomical ambiguity, and technical constraints.

Importantly, much of the existing literature describes fetal sex determination in species-specific terms, making it difficult to extract generalized principles or identify cross-species patterns. Given the author's extensive experience and contributions to fetal sexing in cattle (9), camels (10), buffaloes (11), and horses (15), there is an obvious opportunity to provide a more integrative and translational perspective.

This review therefore aims to bridge the gap between controlled research outcomes and field application by synthesizing biological principles, comparing species-specific characteristics, identifying sources of diagnostic error, and outlining future directions for improving ultrasonographic fetal sex determination in livestock.

## 2 Scope and nature of the review

This article is intended to be a comparative narrative review of embryological, ultrasonographic, and clinical perspectives from various livestock species. The review brings together published evidence with the authors' field and research experience to create a translational framework for practical reproductive management.

## 3 Literature search strategy

A narrative literature search was performed using the PubMed, Scopus, Web of Science, and Google Scholar databases. The literature search included studies published from 1970 to 2026. Keywords used included “fetal sex determination,” “ultrasonography,” “camel,” “cattle,” “horse,” “buffalo,” “genital tubercle,” and “livestock reproduction.” Priority was given to peer-reviewed studies addressing ultrasonographic fetal sexing, embryological development, imaging methodology, comparative species differences, and field applicability in large domestic animals. Classical embryology references as well as recent advances in Doppler imaging, three-dimensional ultrasonography, and artificial intelligence were included. Non-English publications, conference abstracts without sufficient methodological details, and studies unrelated to large domestic animals were excluded.

## 4 Biological basis of fetal sex differentiation

To connect embryological development with clinical ultrasonography, a timeline of external genitalia differentiation across different species is compared (Table 1). Despite interspecies variation, a conserved developmental sequence is visible, beginning with the appearance of the genital tubercle (GT) as an undifferentiated structure and progressing through sex-specific migration and differentiation to definitive external genitalia.

**Table 1:** Comparative timeline of external genitalia development and key diagnostic landmarks for fetal sex differentiation in large domestic animals.

Species	Early stage (GT appearance)	Onset of sexual differentiation	GT migration pattern (Diagnostic basis)	Intermediate development	Advanced differentiation	Fully developed external genitalia	Key diagnostic window (Ultrasound relevance)	References
Horse	40–45 d: GT, urogenital folds, labioscrotal swellings identifiable (indifferent stage)	47–60 d: onset of differentiation	Male: cranial (toward umbilicus); Female: caudal (toward tail)	60–90 d: scrotal/vulvar formation begins; mammary gland development in females	90–120 d: clear scrotum (male); clitoris, vulva, mammary glands (female)	Late gestation: adult-like morphology	~56–120 d (GT-based); extended to 220 d with gonadal/secondary structures	(25, 26, 54)
Camel	35–45 d: GT appears between hind limbs	50–60 d: early differentiation begins	Male: elongation toward penis; Female: clitoral development caudally	60–110 d: penile elongation, scrotal swelling (male); labia, clitoris (female)	110–180 d: clear penis/scrotum (male); vulva/clitoris distinct (female)	200–250 d: fully differentiated genitalia	~65–80 d (narrow GT window); limited later applicability	(55–58)
Cattle	30–35 d: GT and genital swellings visible	55–60 d: clear differentiation detectable	Male: cranial migration; Female: caudal migration	60–90 d: scrotum (male); labia/clitoris (female) clearly visible	>90 d: advanced genital structures resemble adult	>100 d: fully developed genitalia	~56–98 d (optimal and most reliable window)	(19, 35)
Buffalo	38–50 d: GT visible (indifferent stage)	50–60 d: differentiation begins	Male: forward (cranial) elongation; Female: caudal orientation	60–90 d: scrotal swelling (male); vulva, clitoris, teats (female)	90–130 d: distinct penis/scrotum vs. vulva/clitoris	130–180 d: complete differentiation	~70–126 d (moderate feasibility window)	(59–63)

GT, genital tubercle.

Ultrasonographic fetal sexing is based on the identification of genital structures that become distinguishable at specific gestational stages, so accuracy requires a thorough understanding of fetal urogenital anatomy and intra-abdominal positioning. The primordial gonads are undifferentiated and have the potential to develop into testes or ovaries (16–20). The SRY gene largely controls this differentiation, which is influenced by genetic sex as determined by the embryo's karyotype. SRY favors testis formation in XY embryos, but its absence in XX embryos allows for ovarian development (21). The gonads migrate to their final positions during gestation after being initially located near the mesonephros and developing kidneys (16, 19, 21, 22). In the latter half of gestation, with the exception of equines and camelids, male testes descend to the scrotum through the gubernaculum, while female ovaries migrate caudally to the pelvic cavity, with timing and extent differing among species (17, 23). Like equids, camelids demonstrate a comparatively delayed testicular descent in relation to most domestic ruminants and carnivores, with migration typically occurring near birth or within the initial postnatal week (24, 25).

Externally, the genitalia appear on the caudoventral abdominal wall as the GT, genital swellings, and cloacal folds, which later divide into urogenital and anal folds. In males, the GT elongates to form the penis, the urogenital folds form the prepuce, and the genital swellings expand to form the scrotum. In females, the GT forms the clitoris, while the labia develop from genital swellings or urogenital folds (5, 16, 18, 19, 22, 26, 27).

Hormonal regulation by differentiated gonads is required for both internal and external genitalia development. In males, testicular androgens stimulate male genital development, whereas Müllerian duct regression causes the Wolffian ducts to form the epididymis, vas deferens, and accessory glands. In females, the absence of significant androgen influence allows Müllerian ducts to persist and form the oviducts, uterus, and cranial vagina (18, 19, 28–30).

## 5 Ultrasonographic principles and diagnostic landmarks

Two major ultrasonographic approaches are used. Because of its close proximity to the uterus and superior image resolution, the transrectal method is especially effective during early gestation for accurately identifying the GT. In contrast, the transabdominal approach is more appropriate in later stages, when the fetus is deeper within the abdominal cavity (4, 12, 15, 31–33).

The choice of landmark is determined by gestational age, species, and imaging accessibility. Accurate identification necessitates both technical expertise and a thorough knowledge of fetal anatomy. The GT is the primary marker in early gestation, but secondary structures such as the penis, prepuce, scrotum, clitoris, mammary glands, and gonads become more important in mid to late gestation (4, 9, 34). The diagnostic accuracy of these methods is determined not only by the visibility of anatomical landmarks, but also by the operator's ability to correctly interpret them across a variety of fetal orientations and imaging planes. The frontal, cross-sectional, and sagittal views all have distinct advantages depending on fetal positioning and gestational stage (9).

**Figure 1:**
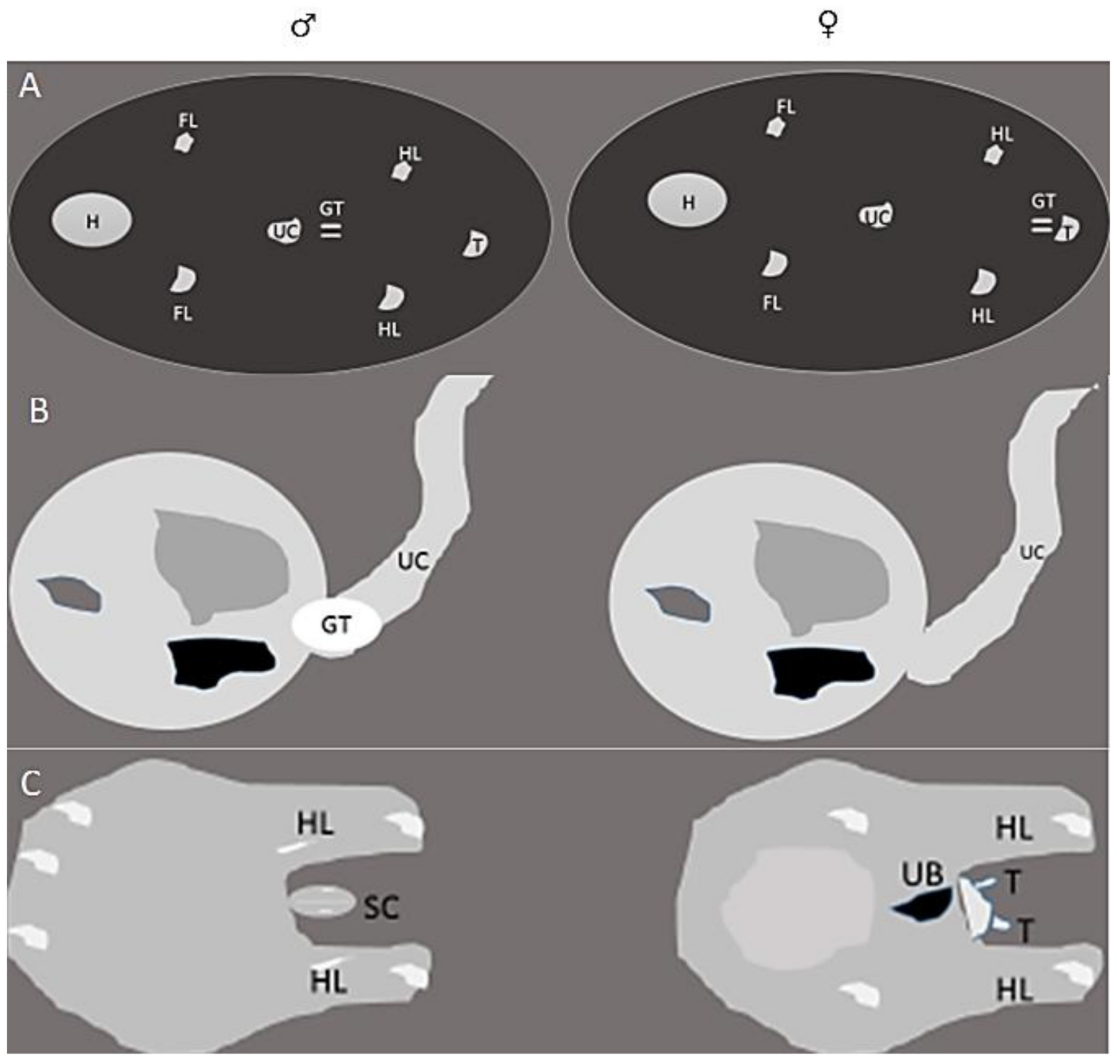
The figure presents schematic illustrations of the ultrasonographic appearance of fetal external reproductive structures in relation to probe orientation and fetal positioning. In the frontal view **(A)**, the transducer is aligned parallel to the fetal body and perpendicular to both the umbilical cord (UC) and the genital tubercle (GT), allowing simultaneous visualization of major anatomical landmarks including the fetal head (H), forelimbs (FR), hind limbs (HL), tail (T), and associated reproductive structures. In the cross-sectional view **(B)**, the transducer is positioned perpendicular to the fetal body as well as to the UC and GT, providing a transverse image that facilitates identification of the GT relative to the umbilical insertion. In the sagittal view **(C)**, the transducer is oriented perpendicular to the body but parallel to the hind limbs, enabling longitudinal assessment of the fetal caudal region and visualization of structures such as the scrotum (SC) and urinary bladder (UB). Together, these imaging planes provide complementary perspectives that enhance the accuracy of fetal sex determination.

**Figure 2:**
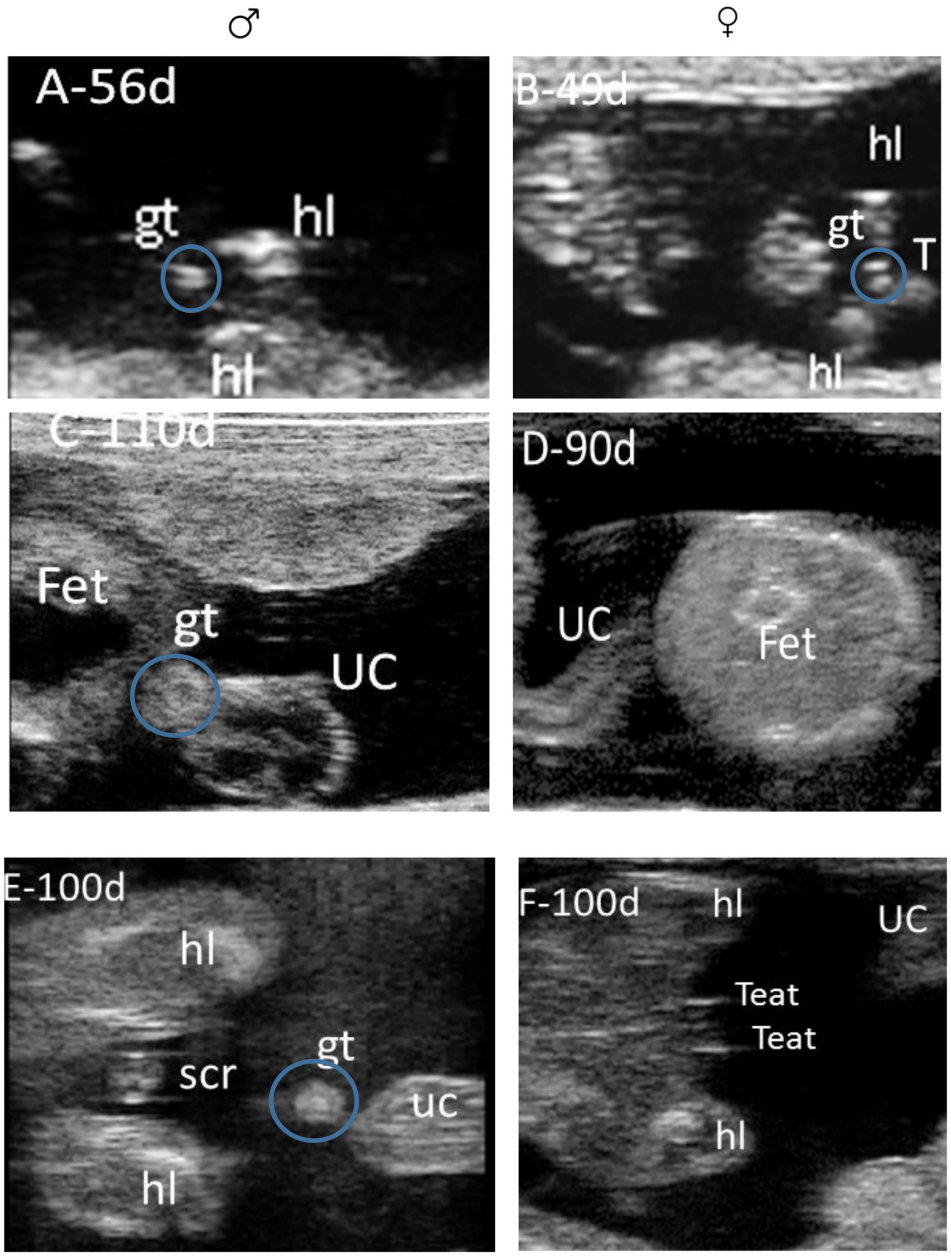
The figure illustrates transrectal ultrasonographic fetal sexing across different imaging planes, including the frontal view **(A, B)**, cross-sectional view **(C, D)**, and sagittal view **(E, F)**. In these images, key anatomical landmarks used for sex determination are identified, including the hind limbs (hl), tail (T), umbilical cord (UC), and genital tubercle (gt), which serves as the primary diagnostic structure during early gestation. Additional features such as the scrotum (scr) and teats become visible at more advanced stages and support sex differentiation when the genital tubercle is less distinct. The fetus (Fet) is visualized in relation to these structures, allowing accurate interpretation of fetal orientation and facilitating identification of sex specific anatomical characteristics across different ultrasonographic planes. **(A–D)** cattle fetuses; **(E, F)** Buffalo fetuses.

The primary goal of transrectal fetal gender determination is to identify the GT. During development, the GT moves from its initial position between the caudal limbs to the umbilical cord in males and the tail in females (19, 25, 35, 36). A real-time B-mode ultrasound scanner with a linear array transducer capable of 5–7 MHz is required for this method. Before inserting the probe at the uterine level, the animal should be properly restrained and any feces removed from the rectum. The fetal sex can be determined using three sonographic planes: frontal, cross-sectional, and sagittal (Figures 1A–C). The examiner can choose a view based on fetal movement or by adjusting the transducer (9). In the frontal view (Figure 1A), the transducer is positioned to capture the fetal head, forelimbs, umbilical cord, hind limbs, and tail all in one frame. The GT is positioned between the umbilical cord and the tail. In the cross-sectional view (Figure 1B), the probe is placed transversely across the fetus, beginning at a reference point such as the head or beating heart and progressing caudally to the umbilical insertion. In males, the GT is a hyperechoic bilobed structure located just behind the umbilical cord. In females, the scanning extends to the hind limbs and tail, where the GT is visible beneath the tail. In the sagittal view (Figure 1C), the fetus is imaged longitudinally to measure the distance between the hind limbs. This allows the visualization of the scrotum in male fetuses and the mammary glands in female fetuses. According to the authors' experience, the frontal view is the most straightforward to obtain during this gestational period. However, Renaudin et al. (31) found that the cross-sectional view is more accessible and easier to understand. If none of these views can be clearly obtained within 10 min, the examination should be stopped and repeated when the fetal positioning is more favorable. Figure 2 depicts examples of how these three imaging planes can be used to identify and interpret external genitalia.

**Figure 3:**
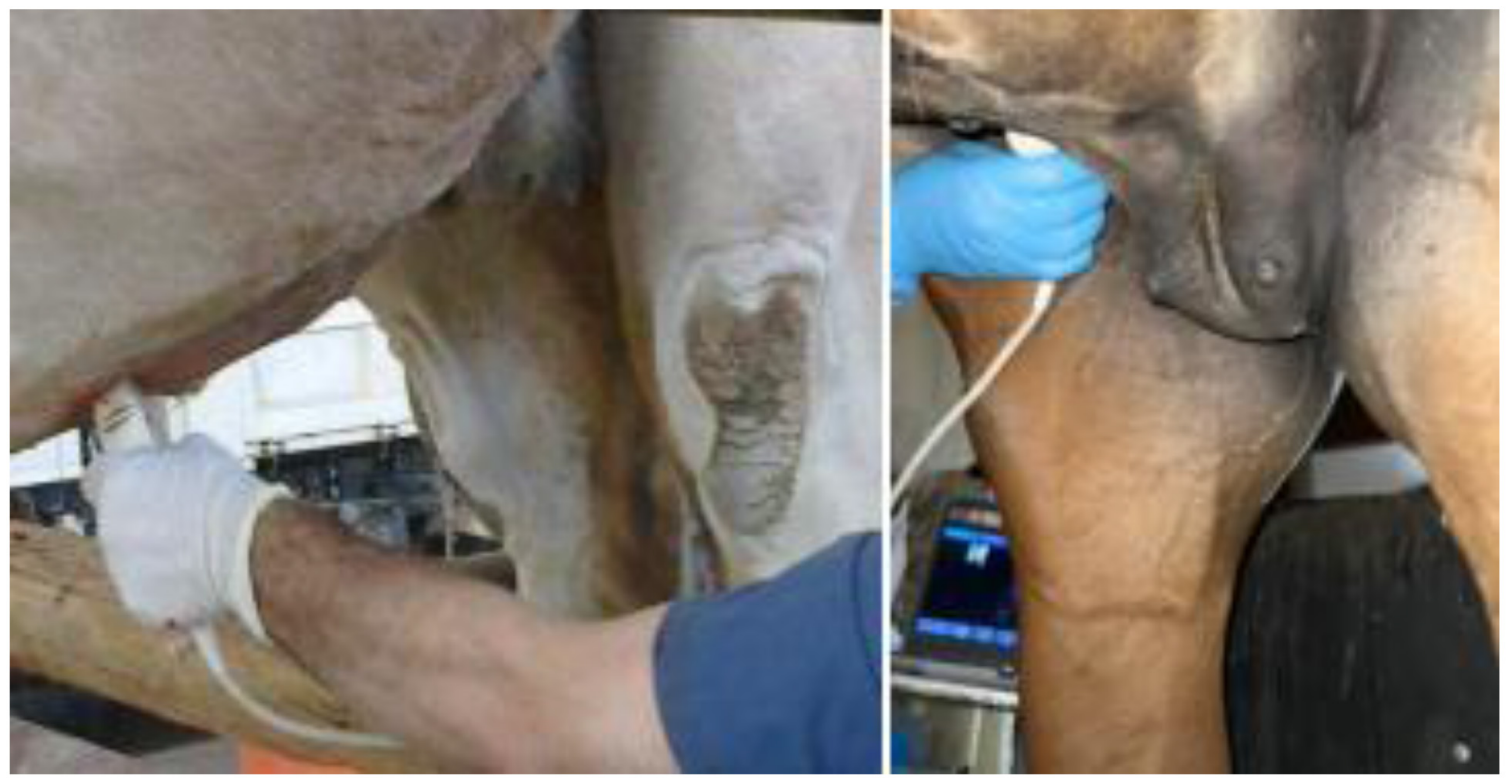
Transabdominal ultrasonographic examination in a mare **(right)** and a female dromedary camels **(left)** using curvilinear transducer and ultrasound gel. The examination starts by placing the transducer on the ventral midline, cranial to the mammary glands. Fetal fluids and parts are often visible here; if not, the probe is moved laterally. In younger fetuses located deeper in the abdomen, the transducer may be positioned in the inguinal region above the mammary glands. Once the fetus is located, scanning is guided by fetal anatomy, with emphasis on visualizing the hindquarters.

Animals are typically kept in stocks to determine transabdominal fetal gender (Figure 3). Hair can be clipped from the mammary glands to the xiphoid region, with lateral extensions on both sides. The area is then cleansed with water and alcohol. A high-quality ultrasonographic coupling gel is then applied to ensure that the probe has the best possible contact with the skin. A real-time B-mode diagnostic ultrasound scanner with linear or curvilinear probes ranging in frequency from 3.5 to 5.0 MHz is required. The 5 MHz linear probe is suitable for mid-gestation fetuses, whereas the 3.5 MHz probe is preferable for later gestation due to its greater penetration. Many practitioners prefer linear probes because they have a larger contact surface (footprint) and allow for better spatial orientation by less experienced ultrasonographers (31, 37). The transducer is placed along the ventral midline, just cranial to the mammary glands (Figure 3). Fetal parts and fluids are frequently visible in this area, which is just dorsal to the uterus and placenta. If these structures are not visible, position the transducer laterally away from the midline. For younger fetuses located deeper in the abdominal cavity, the probe may be placed in the inguinal region, directly above the mammary glands. After identifying the fetus, the transducer is guided over the abdomen in accordance with fetal anatomy, with the primary goal of visualizing the hindquarters (31, 32, 37–39).

**Figure 4:**
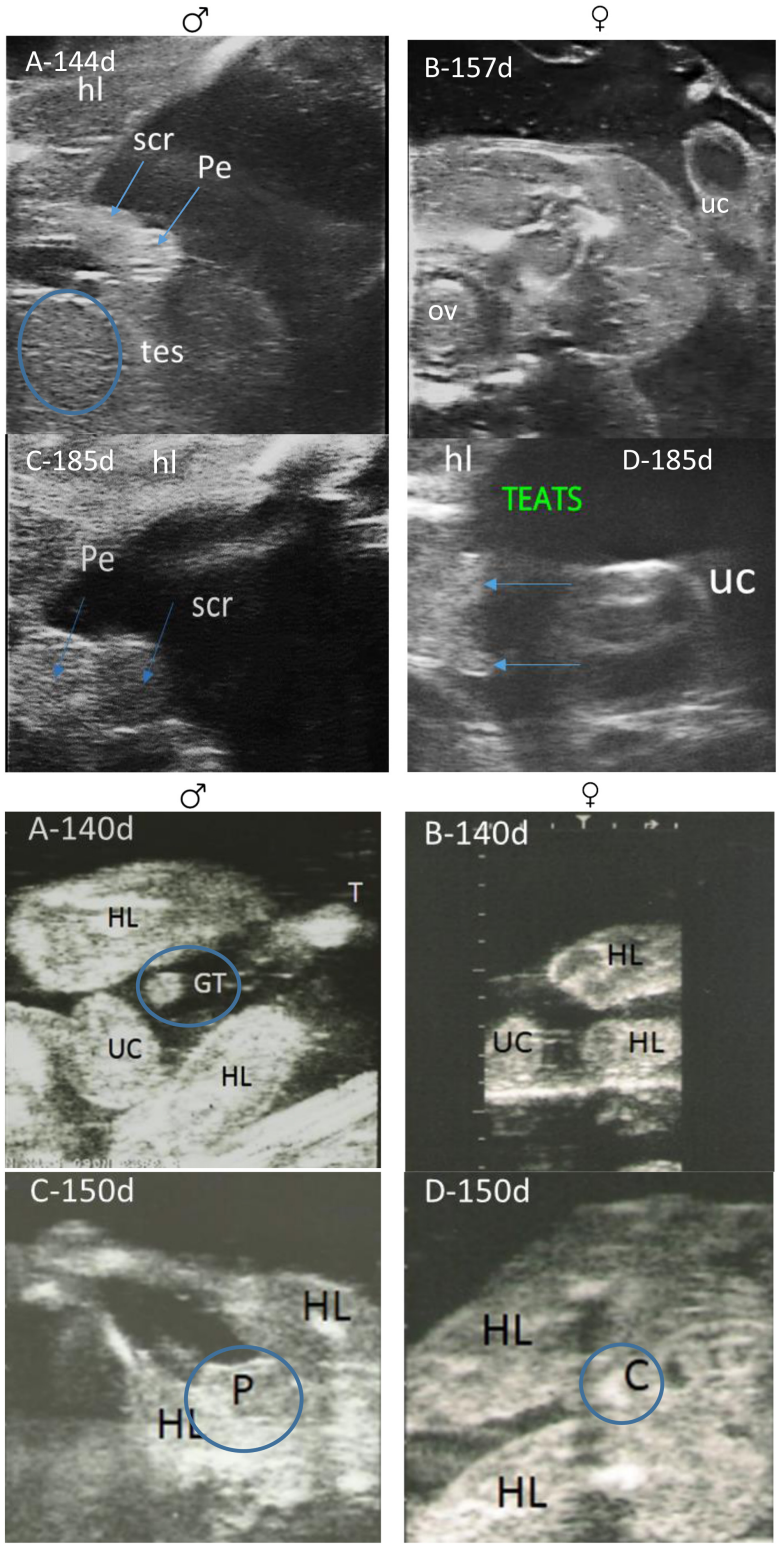
Ultrasonographic identification of fetal sex in equine pregnancy during mid- to late gestation. In male fetuses, the testes (tes) are visualized in the caudal abdomen as homogeneous, echogenic, rounded structures, while the scrotum (scr) and penis (Pe) are identified between the hind limbs **(A, C)**. In female fetuses, the ovaries (ov) appear as oval, relatively less echogenic structures surrounded by a hypoechoic circumscribed zone, whereas the mammary glands (Mam) and teats are observed between the hind limbs (hl) **(B, D)**. uc, umbilical cord.

**Figure 5:**
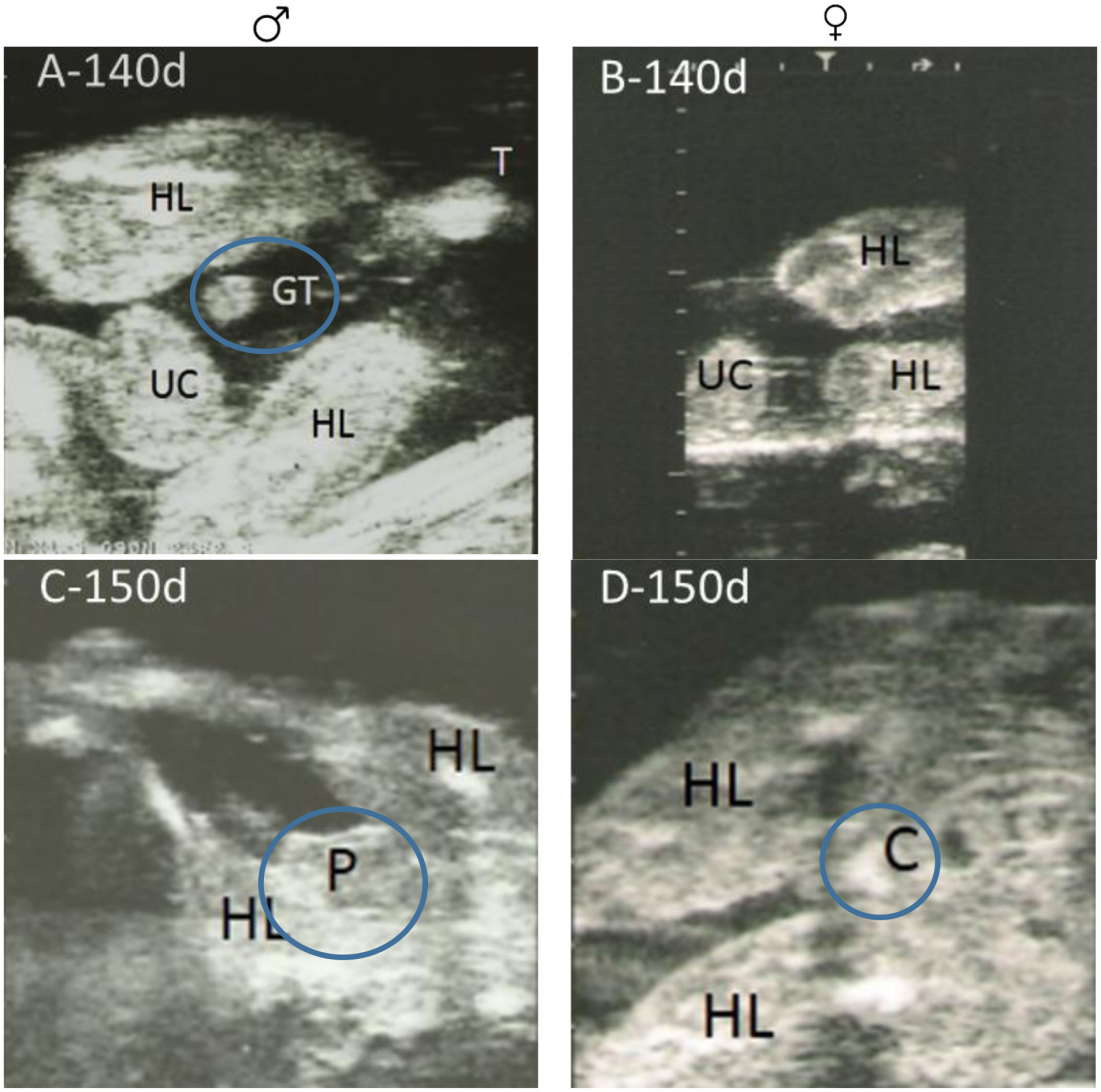
Ultrasonographic differentiation of male and female camel fetuses. In cross-sectional view, the genital tubercle (GT) is located between the hind limbs near the umbilical cord in males **(A)** but is not visible in this position in females **(B)**. In sagittal view, the male prepuce appears as a characteristic triangular structure between the hind limbs **(C)**, whereas in females, the clitoris is seen beneath the tail as a relatively hypoechoic structure **(D)**.

As gestation progresses, a combined transrectal and transabdominal ultrasonographic approach is recommended, particularly in equine practice, to better visualize secondary sexual structures such as the penis/prepuce, gonads, and mammary glands. During this stage, the scrotum can be distinguished between the hind limbs in male fetuses, whereas the clitoris is visualized in female fetuses (15, 31, 40) (Figure 4). The GT is visible in the cross-sectional view of the male camel fetus, located between the hind limbs and close to the umbilical cord, whereas it is not visible in female fetuses. In the sagittal plane, the male fetus is distinguished by the presence of the prepuce, a distinct triangular structure between the hind limbs. In contrast, in the female fetus, the clitoris is found directly beneath the tail as a more hypoechoic structure (Figure 5).

## 6 Comparative analysis across species

In this review, feasibility is defined as the practical likelihood of consistently obtaining diagnostically interpretable images under routine field conditions, taking into account fetal accessibility, imaging window duration, fetal mobility, and operator dependency. To facilitate cross-species comparison, feasibility was categorized as high, moderate, or low based on a combination of practical criteria, including the duration of the diagnostic window, ease of visualization of diagnostic landmarks, consistency under field conditions, requirement for repeated examinations, and degree of operator dependency. Importantly, feasibility in this review refers to the practical ability to consistently obtain and interpret diagnostic images under routine clinical conditions rather than diagnostic accuracy alone. Accordingly, cattle showed the highest feasibility due to stable fetal positioning and consistent GT visualization, whereas horses demonstrated moderate feasibility because of increased fetal mobility and greater operator dependency. Camels and buffaloes showed lower feasibility due to narrower diagnostic windows, deeper fetal positioning, and limited imaging accessibility under field conditions.

The comparative synthesis presented in Table 2 demonstrates that ultrasonographic fetal sex determination in large domestic animals is limited not by diagnostic accuracy, which consistently exceeds 90% under optimal conditions, but by species-specific embryological timing, anatomical accessibility, fetal mobility differences, uterine positioning, and operator-dependent limitations. This distinction is critical because it shifts the focus away from methodological capability and toward biological and practical constraints, which ultimately determine field applicability.

**Table 2:** Comparative overview of ultrasonographic fetal sex determination in large domestic animals.

Species	Main technique(s)	Key diagnostic landmark(s)	Reported feasibility (%)	Reported accuracy (%)	Optimal window (Days)	Extended diagnostic window	Key limitations	Representative references
Horse	Transrectal (early); Transabdominal (mid–late); Doppler (adjunct)	Genital tubercle (GT), gonads, penis/prepuce, mammary gland	65–100	81.8–100	105–133 (GT); 90–180 (gonads)	Up to 220–270 (transabdominal)	High fetal mobility; variable positioning	(4, 7, 10, 13–15, 31, 32)
Camel	Transrectal (B-mode)	GT	51–86.5	91–96.9	~70–77	Limited beyond 150 days (transrectal)	Narrow diagnostic window; limited studies	(4, 10)
Cattle	Transrectal (B-mode)	GT, scrotum, testes, mammary gland	55.6–100	92–100	56–98	Up to ~120 days (reduced reliability)	Fetal position dependency	(9, 41, 42)
Buffalo	Transrectal (B-mode)	GT, scrotum, testes, mammary gland	63–64.8	64–97.1	70–126	Declines after ~150 days	Lower feasibility; limited data	(11, 43)

Feasibility reflects the practical ease of fetal sex determination under field conditions and was estimated based on diagnostic window duration, ease of landmark visualization, fetal accessibility, need for repeated examinations, operator dependency, and consistency of results across studies. Reported accuracy represents the percentage of correctly predicted fetal sexes relative to the confirmed fetal sex at birth or later gestation in the referenced studies.

GT, genital tubercle.

The GT plays a critical role as the primary diagnostic landmark during early gestation, which is a common feature across all species. As gestation advances, reliance progressively shifts from the GT to secondary landmarks such as the scrotum, prepuce, mammary glands, gonads, and external genitalia. The visibility of these structures is influenced not only by species-specific developmental timelines but also by factors including fetal orientation, imaging angle, uterine depth, operator expertise, and ultrasound equipment quality. These differences emphasize the importance of adapting diagnostic strategies to the biological and practical characteristics of each species rather than applying a uniform protocol across all livestock species (15, 16, 19, 22).

Across species, cattle are the most consistent and predictable model for early fetal sex determination. The relatively stable intrauterine position of the bovine fetus, combined with the clear and early migration pattern of the GT, allows for reliable diagnosis within a well-defined window of days 56 to 98 of gestation (5, 8, 9, 33, 41, 42). This high level of feasibility and accuracy can be attributed to favorable anatomy and extensive field validation in dairy production systems.

Buffaloes, on the other hand, exhibit lower feasibility and greater variability, most likely due to differences in fetal positioning and a scarcity of comprehensive studies (11, 43). This discrepancy highlights the importance of species-specific optimization rather than direct extrapolation from bovine models.

The clinical approach and anatomical reference points in equine practice differ significantly. When combining transrectal and transabdominal approaches, horses provide the most comprehensive diagnostic window of any species reviewed (7, 15, 31, 32, 40). However, this advantage is balanced out by increased technical complexity. High fetal mobility during early gestation frequently complicates consistent visualization of the GT, necessitating increased operator experience and, in some cases, repeated examinations (15, 22, 34, 44). The addition of Doppler ultrasonography has increased diagnostic confidence, especially during mid-gestation when gonadal structures become more visible (13, 14, 45, 46). In addition, advanced imaging approaches such as three-dimensional ultrasonography have further enhanced diagnostic performance in equine reproduction (37, 40, 46, 47).

Camelids pose a distinct diagnostic challenge. Despite achieving high accuracy rates comparable to other species, the feasibility of fetal sex determination in camels remains limited, with a narrow gestational window centered on days 70 to 77 (4, 10). This limitation is primarily due to the rapid transition of the GT and the difficulty of maintaining optimal imaging conditions after early gestation using transrectal techniques. Furthermore, the scarcity of studies and the limited use of complementary approaches, such as transabdominal ultrasound for sex determination, limit diagnostic flexibility. These findings highlight a critical gap in the literature, emphasizing the importance of methodological expansion tailored specifically to camel reproduction.

A cross-species comparison (Table 2) reveals that while ultrasonographic fetal sexing achieves high accuracy (>90%) across most species, feasibility and optimal diagnostic windows are highly species-dependent. Horses offer the widest diagnostic window due to the complementary use of transrectal and transabdominal approaches, whereas camels present a narrower and more challenging window despite comparable accuracy. In contrast, cattle provide the most consistent and predictable conditions for early diagnosis, while buffaloes remain less explored with relatively lower feasibility.

## 7 Determinants of diagnostic success

Despite favorable conditions, several factors can have a significant impact on diagnostic success (Table 3). Operator expertise and fetal positioning are the most important determinants, accounting for many of the differences between controlled studies and field applications (7–9, 31, 32, 36).

**Table 3:** Key factors affecting accuracy of fetal sexing.

Factor	Impact on diagnosis
Fetal position	Obscures landmarks
Operator experience	Major determinant of accuracy
Equipment quality	Affects resolution and depth
Gestational age	Determines landmark visibility
Species differences	Alters anatomical interpretation

Misdiagnosis in fetal sex determination can occur due to confusion between the GT and adjacent structures (e.g., umbilical cord), suboptimal imaging angles, and excessive fetal movement (4, 10, 11, 31, 33, 34, 44). Recognizing these potential sources of error is critical for increasing diagnostic reliability and developing standardized protocols.

Species-specific anatomical and developmental variations substantially contribute to diagnostic challenges in ultrasonographic fetal sex determination, particularly when criteria established for one species are extrapolated to another. Operator experience, learning curves, probe frequency, image resolution, and differences between portable and high-end ultrasound systems are other practical factors that may limit the consistency of ultrasonographic fetal sex determination under field conditions. Environmental and handling conditions may further affect image quality and fetal accessibility. These limitations highlight the importance of standardized operator training and optimization of imaging protocols to improve diagnostic reproducibility across species and production systems.

## 8 Practical implications

Under field conditions, feasibility is the most important limiting factor, not accuracy. While clear visualization of diagnostic landmarks can result in high accuracy, the ability to obtain such images consistently is dependent on fetal position, gestational age, operator expertise, and equipment quality (4, 5, 8, 9, 13, 14, 42, 48). This is especially evident in species such as buffaloes and camels, where lower feasibility rates reduce the technique's overall effectiveness despite satisfactory accuracy in successful cases (4, 10, 11). As a result, future improvements should prioritize improving imaging accessibility and consistency rather than simply refining diagnostic criteria.

From a clinical standpoint, successful fetal sex determination necessitates careful selection of the appropriate gestational window, species-specific diagnostic criteria, and optimal imaging technique. Adequate animal handling, proper equipment selection, and ongoing operator training are required to maximize diagnostic accuracy (4, 5, 13–15, 33, 37).

The comparative framework emphasizes the importance of species-specific guidelines for effective implementation. In cattle, a single well-timed transrectal examination is frequently sufficient, whereas in horses, a combination of approaches and possibly repeated scans may be required (5, 7, 9, 32, 33, 46). To maximize success in camels and buffaloes, precise timing within a narrow diagnostic window is required (4, 10, 11). These distinctions have direct implications for reproductive management strategies, economic decision-making, and the incorporation of fetal sexing into standard veterinary practice (1, 2, 15, 37).

Operator experience and ultrasound equipment quality are also major determinants of diagnostic success in fetal sex determination. Accurate identification of the genital tubercle and secondary genital structures requires detailed anatomical knowledge, species-specific experience, and appropriate imaging skills. Diagnostic accuracy generally improves with operator training and field experience, particularly in species with high fetal mobility such as horses and camels (4, 8, 44, 48). Equipment-related factors, including probe frequency, image resolution, and Doppler capability, also influence visualization quality and diagnostic feasibility. In addition, field conditions such as animal restraint and examination environment may affect imaging consistency. These factors highlight the importance of standardized training protocols and species-specific ultrasonographic guidelines to improve reproducibility under practical field conditions.

Translating controlled findings into realistic field protocols that account for variability in animals, environments, and operator expertise will be critical for closing the research-practice gap.

## 9 Artificial intelligence

Artificial intelligence (AI), particularly deep learning, is emerging as an exciting tool in ultrasonographic imaging, with potential applications in fetal sex determination. Convolutional neural networks (CNNs) have achieved high accuracy (>95%) in automated fetal image classification and anatomical recognition, frequently matching expert performance (49–51). AI has been used successfully in human obstetrics to detect fetal structure, estimate gestational age, and assess image quality (51–53). Although direct applications in livestock fetal sexing are limited, these advances indicate significant translational potential. AI-assisted systems have the potential to reduce operator dependency, improve diagnostic consistency, and increase field feasibility. However, progress in veterinary applications will depend on the availability of large, annotated, species-specific datasets and robust validation in real-world environments.

## 10 Knowledge gaps and research priorities

Despite significant progress, there are still several gaps in our current understanding and application of fetal sex determination. Transabdominal ultrasonography has not been thoroughly investigated for fetal sexing in camels and buffaloes, suggesting a potential area for future research. Similarly, the integration of Doppler ultrasonography, three-dimensional imaging, and artificial intelligence remains limited in large animal practice.

Another critical gap is the lack of standardized protocols that take into account species differences, operator variability, and field conditions. Furthermore, large-scale validation studies under commercial farming conditions are scarce, limiting the applicability of existing findings.

## 11 Conclusion

The comparative analysis shows that, while ultrasonographic fetal sex determination is a highly accurate tool for large domestic animals, its practical success is heavily influenced by species-specific biological and technical factors. Recognizing and addressing these differences is essential for optimizing its use and advancing reproductive management in diverse livestock systems. Expanding the use of transabdominal ultrasonography in camels and buffaloes, integrating Doppler and three-dimensional imaging across species, and investigating automated or machine learning-assisted image interpretation are all promising ways to improve feasibility and reproducibility. Such advancements would not only improve diagnostic performance but also reduce operator dependency, facilitating wider adoption in field conditions.

## References

[1] Fricke PM. Scanning the future – ultrasonography as a reproductive management tool for dairy cattle. J Dairy Sci. (2002) 85:1918–26. doi: 10.3168/jds.S0022-0302(02)74268-9 12214984

[2] Aurich C Schneider J. Sex determination in horses - current status and future perspectives. Anim Reprod Sci. (2014) 146:34–41. doi: 10.1016/j.anireprosci.2014.01.014 24598214

[3] Kim D Son M Jung D Heo S Kim M Yi J. Economic impacts of ultrasonographic fetal sex determination on Hanwoo cattle profitability and market dynamics. Vet Sci. (2025) 12:201. doi: 10.3390/vetsci12030201 40266948 PMC11946265

[4] Nasef M. Fetal sex determination in dromedary camels in the first trimester using trans-rectal ultrasonography. Reprod Domest Anim. (2026) 61:e70193. doi: 10.1111/rda.70193 41952546

[5] López-Gatius F Garcia-Ispierto I. Sexing of embryos at the time of twin reduction: a clinical approach. Animals. (2023) 13:1326. doi: 10.3390/ani13081326 37106889 PMC10134968

[6] McKimmie C Forutan M Tajet HM Ehsani A Hickford J Amirpour H. Impact of implementing female genomic selection and the use of sex-selected Semen technology on genetic gain in a dairy herd in New Zealand. Int J Mol Sci. (2025) 26:990. doi: 10.3390/ijms26030990 39940759 PMC11817307

[7] Curran S Ginther OJ. Ultrasonic diagnosis of equine fetal sex by location of the genital tubercle. J Equine Vet Sci. (1989) 9:77–83. doi: 10.1016/S0737-0806(89)80032-2

[8] Curran S Ginther OJ. Ultrasonic determination of fetal gender in horses and cattle under farm conditions. Theriogenology. (1991) 36:809–14. doi: 10.1016/0093-691X(91)90346-F 16727049

[9] Ali A. Effect of gestational age and fetal position on the possibility and accuracy of ultrasonographic fetal gender determination in dairy cattle. Reprod Domest Anim. (2004) 39:190–4. doi: 10.1111/j.1439-0531.2004.00502.x 15182296

[10] Ali A Al-Sobayil F Derar R El-Tookhy O. Ultrasonographic fetometry and prenatal fetal sex assessment in camels (*Camelus dromedarius*). Theriogenology. (2013) 80:609–18. doi: 10.1016/j.theriogenology.2013.05.028 23830233

[11] Ali A Fahmy S. Ultrasonographic fetometry and determination of fetal sex in buffaloes (*Bubalus bubalis*). Anim Reprod Sci. (2008) 106:90–9. doi: 10.1016/j.anireprosci.2007.04.010 17544605

[12] Criṣan MI Damian A Morar I Páll E Peṣtean C Groza IṢ. Equine embryo sexing and ultrasonographic foetal sexing – interests and applicability. Anat Histol Embryol. (2016) 45:329–37. doi: 10.1111/ahe.12205 26424663

[13] Resende HL Carmo MT Alvarenga MA. Use of Doppler ultrasound for equine fetal sex determination. Reprod Fertil Dev. (2012) 25:285. doi: 10.1071/RDv25n1Ab274

[14] Resende HL Carmo MT Ramires Neto C Alvarenga MA. Determination of equine fetal sex by Doppler ultrasonography of the gonads. Equine Vet J. (2014) 46:756–8. doi: 10.1111/evj.12213 24237116

[15] Ali A Derar DR Alaeyeari AA Alharbi YM. Fetometry in Arabian horses. Front Vet Sci. (2025) 12:1689769. doi: 10.3389/fvets.2025.1689769 41209474 PMC12588862

[16] Noden DM De Lahunta A. The Embryology of Domestic Animals. Baltimore, MD: Williams and Wilkins (1985).

[17] Dyce KM Sack WO Wensing CJG. Textbook of Veterinary Anatomy, 4th Edn. Amsterdam: Saunders Elsevier (2010).

[18] Hyttel P Sinowatz F Vejlsted M. Essentials of Domestic Animal Embryology. Amsterdam: Saunders Elsevier (2010).

[19] McGeady TA Quinn PJ FitzPatrick ES Ryan MT Kilroy D. Veterinary Embryology, 2nd Edn. Hoboken, NJ: Wiley-Blackwell (2017).

[20] Lamothe S Bernard V Christin-Maitre S. Gonad differentiation toward ovary. Ann Endocrinol. (2020) 81:83–8. doi: 10.1016/j.ando.2020.04.004 32340851

[21] Nagahama Y Chakraborty T Paul-Prasanth B Ohta K Nakamura M. Sex determination, gonadal sex differentiation, and plasticity in vertebrate species. Physiol Rev. (2021) 101:1237–308. doi: 10.1152/physrev.00044.2019 33180655

[22] Ginther OJ. Reproductive Biology of the Mare: Basic and Applied Aspects, 2nd Edn. Cross Plains, WI: Equiservices Publishing (1992).

[23] Hutson JM Nation T Balic A Southwell BR. The role of the gubernaculum in the descent and undescent of the testis. Ther Adv Urol. (2009) 1:115–21. doi: 10.1177/1756287209105266 21789060 PMC3126055

[24] Tibary A Anouassi A. Theriogenology in Camelidae: Anatomy, Physiology, Pathology and Artificial Breeding. Arles: Actes Éditions (1997).

[25] McKinnon AO Squires EL Vaala WE Varner DD. Equine Reproduction, 2nd Edn. Hoboken, NJ: Wiley-Blackwell (2011).

[26] Evans HE Sack WO. Prenatal development of domestic and laboratory mammals: growth curves, external features, and selected references. Anat Histol Embryol. (1973) 2:11–45. doi: 10.1111/j.1439-0264.1973.tb00253.x 4745140

[27] Cunha GR Baskin LS. Development of the external genitalia. Differentiation. (2020) 112:7–9. doi: 10.1016/j.diff.2019.10.008 31881402 PMC7138693

[28] Di Berardino D Iannuzzi L. Cytogenetics of camelidae. In: Cytogenetics of the Domestic Animals. Wallingford: CABI Publishing (2003). p. 317–49.

[29] Noakes DE Parkinson TJ England GCW. Arthur's Veterinary Reproduction and Obstetrics, 9th Edn. Amsterdam: Elsevier (2018).

[30] Holterhus PM Kulle A Busch H Spielmann M. Classic genetic and hormonal switches during fetal sex development and beyond. Med Genet. (2023) 35:163–71. doi: 10.1515/medgen-2023-2036 38840820 PMC10842585

[31] Renaudin CD Gillis CL Tarantal AF. Transabdominal ultrasonographic determination of fetal gender in the horse during mid-gestation. Equine Vet J. (1999) 31:483–7. doi: 10.1111/j.2042-3306.1999.tb03855.x 10596929

[32] Van de Velde M Heidbuchel M Beert L Wydooghe E Van Soom A. Ultrasonographic fetal sex determination in horses: a practical guide. Equine Vet Educ. (2018) 30:511–9. doi: 10.1111/eve.12808

[33] Bogdan LM Petrean AB Coman I Nadă? G Cenariu M Bogdan I . The diagnosis of fetal sexing in cattle using ultrasound. Bull Univ Agric Sci Vet Med Cluj Napoca Vet Med. (2019) 76:149–53. doi: 10.15835/buasvmcn-vm:2019.0008

[34] Bucca S. Equine fetal gender determination from mid- to advanced-gestation by ultrasound. Theriogenology. (2005) 64:568–71. doi: 10.1016/j.theriogenology.2005.05.013 15993481

[35] Inomata T Eguchi Y Yamamoto M Asari M Kano Y. Development of external genitalia in bovine fetuses. Japan J Vet Sci. (1982) 44:489–96. doi: 10.1292/jvms1939.44.489 7132019

[36] Rasheed YM Khalaf FM Mohammed SN. Assessment of fetal sex determined and eye diameter to detection of gestational age in mares by ultrasonography. Iraqi J Vet Sci. (2023) 37:129–34. doi: 10.33899/ijvs.2023.1375080.2691

[37] Bollwein H Pricking S Spilker K Martinsson G Rau J Toenissen A. Equine fetal gender determination in mid- and advanced gestation by transabdominal approach: comparative study using 2D B-mode ultrasound, Doppler sonography, 3D B-mode, and following tomographic ultrasound imaging. Pferdeheilkunde. (2019) 35:11–9. doi: 10.21836/PEM20190102

[38] Ali A Derar R Al-Sobayil F. Transabdominal ultrasonography for pregnancy diagnosis and estimation of gestational age in dromedary camels. Reprod Dom Anim. (2015) 50:437–42. doi: 10.1111/rda.12510 25800152

[39] Ali A Derar DR Abdel-Razek AK. Ultrasonography for the detection of pregnancy and study of embryonic and fetal development in camels, buffaloes, and sheep: techniques, equations, and limitations. Anim Reprod Sci. (2024) 268:107566. doi: 10.1016/j.anireprosci.2024.107566 39089168

[40] Neal H. Determining the sex of equine fetuses: an update. In Pract. (2019) 41:34–9. doi: 10.1136/inp.k4996

[41] Müller E Wittkowski G. Visualization of male and female characteristics of bovine fetuses by real-time ultrasonics. Theriogenology. (1986) 25:571–4. doi: 10.1016/0093-691X(86)90140-8 16726147

[42] Quintela LA Becerra JJ Pérez-Marín CC Peña AI Herradón PG Prieto A. Fetal gender determination by first-trimester ultrasound in dairy cows under routine herd management in Northwest Spain. Anim Reprod Sci. (2011) 125:13–9. doi: 10.1016/j.anireprosci.2011.02.022 21398058

[43] Yotov S Atanasov A Georgiev P. Determination of foetal sex in buffaloes through a single sonographic examination. Bulg J Vet Med. (2011) 14:39–44.

[44] Tönissen A Martinsson G Pricking S Otzen H Ertmer F Rau J . Transabdominal ultrasonographic determination of fetal gender in the horse during mid-gestation: a comparative study using randomized video images to investigate variation in diagnostic performance among raters, and the effect of month of gestation. Pferdeheilkunde. (2016) 32:29–35. doi: 10.21836/PEM20160105

[45] Mebarki M Kaidi R Azizi A Basbaci M. Comparative efficacy of two-dimensional mode and color Doppler sonography in predicting gender of the equine fetus. Vet World. (2019) 12:325–30. doi: 10.14202/vetworld.2019.325-330 31040577 PMC6460860

[46] Becsek A Schweizer A Knutti B Bollwein H. Gender determination in equine fetuses in early pregnancy using two- and three-dimensional ultrasound. Tierärztl Prax. (2020) 48:166–71. doi: 10.1055/a-1161-9797 32557511

[47] Kotoyori Y Yokoo N Ito K Murase H Sato F Korosue K . Three-dimensional ultrasound imaging of the equine fetus. Theriogenology. (2012) 77:1480–6. doi: 10.1016/j.theriogenology.2011.10.020 22192400

[48] Mari G Castagnetti C Belluzzi S. Equine fetal sex determination using a single ultrasonic examination under farm conditions. Theriogenology. (2002) 58:1237–43. doi: 10.1016/S0093-691X(02)00943-3 12240926

[49] Shen D Wu G Suk HI. Deep learning in medical image analysis. Annu Rev Biomed Eng. (2017) 19:221–48. doi: 10.1146/annurev-bioeng-071516-044442 28301734 PMC5479722

[50] Kaplan E Ekinci T Kaplan S Barua PD Dogan S Tuncer T . PFP-LHCINCA: pyramidal fixed-size patch-based feature extraction and chi-square iterative neighborhood component analysis for automated fetal sex classification on ultrasound images. Contrast Media Mol Imaging. (2022) 2022:6034971. doi: 10.1155/2022/6034971 35655731 PMC9132621

[51] Benson M Walton S Hartley T Meagher S Seshadri S Sleep N . Fetal gestational age estimation using artificial intelligence on non-targeted ultrasound images and video. NPJ Digit Med. (2025) 8:700. doi: 10.1038/s41746-025-02024-z 41266569 PMC12635104

[52] Horgan R Nehme L Abuhamad A. Artificial intelligence in obstetric ultrasound: a scoping review. Prenat Diagn. (2023) 43:1176–219. doi: 10.1002/pd.6411 37503802

[53] Frisch EH Jain A Jin M Duhaime EP Malshe A Corey S . Artificial intelligence to determine fetal sex. Am J Perinatol. (2024) 41:1836–40. doi: 10.1055/a-2265-9177 38336117

[54] Franciolli ALR Cordeiro BM Fonseca ET Rodrigues MN Sarmento CAP Ambrosio CE . Morphogenesis of the external genitalia in equine fetuses. Anat Histol Embryol. (2011) 40:441–6. doi: 10.1111/j.1439-0264.2011.01095.x21843210

[55] Osman DI El-Wishy AB. Development of the genital organs in the camel fetus (*Camelus dromedarius*). Br Vet J. (1981) 137:527–37.7306781

[56] El-Maghawry AM Fahmy M. Prenatal development of genitalia in camel (*Camelus dromedarius*). Camel Newslett. (1995) 11:11–5.

[57] Ismail ST Abdel-Raouf M. Studies on prenatal development of reproductive system in camels. J Camel Pract Res. (2005) 12:131–5.

[58] Oyelowo FO Sonfada ML Umar AA Abubakar MS Danmaigoro A Atabo MS . Pre-natal morphology of male reproductive organs of *Camelus dromedarius*. *J Adv Vet Res*. (2022) 12:480–4.

[59] Abdel-Raouf M El-Naggar MA. Further study of the biometry and development of the Egyptian buffalo foetus. United Arab J Vet Sci. (1970) 7:125–40.

[60] Abdel-Raouf M El-Naggar MA Fateh El-Bab MR. The development of the fetal testis in the buffalo. Z Anat Entwicklungsgesch. (1974) 144:227–35. doi: 10.1007/BF00519778 4416158

[61] Sarma K Bhaskar TVS Rao GS. Morphogenesis of the external genitalia in buffalo foetuses. Indian J Anim Sci. (2002) 72:1002–5.

[62] Shukla SP Singh Y Singh I. Histogenesis and morphogenesis of the gonads and external genitalia in buffalo foetuses. Indian J Anim Sci. (2007) 77:125–9.

[63] Ali A Abdel-Raouf M Ahmed WM. Prenatal development of genital organs in buffalo with reference to fetal sexing. Glob Vet. (2010) 5:219–26.

